# Reno-Gonadal Venous Anastomosis for Right Renal Venous Drainage Following Juxtarenal Cavectomy for Renal Cell Carcinoma

**DOI:** 10.15586/jkc.v12i2.390

**Published:** 2025-04-21

**Authors:** Jabrina Simmons, Tae-Hee Kim, Tasha Posid, Shawn Dason

**Affiliations:** Division of Urologic Oncology, The Ohio State University Comprehensive Cancer Center, Columbus, OH

**Keywords:** inferior vena cava tumor thrombus, juxtarenal cavectomy, renal cell carcinoma, reno-gonadal drainage, vascular reconstruction

## Abstract

This article is a case report of a 62-year-old male with a left-sided renal cell carcinoma (RCC) with a level II inferior vena cava (IVC) thrombus and caval occlusion. He was managed with open left radical nephrectomy and juxtarenal cavectomy. To preserve right renal venous drainage, the right renal vein was anastomosed to the right gonadal vein. He has not had any renal functional decline or disease recurrence with 3 years of follow-up. The focus of this article is to discuss this distinctive method for vascular reconstruction as an option for right renal venous drainage following left nephrectomy and juxtarenal cavectomy.

## Introduction

Renal cell carcinoma (RCC) has the tendency to invade contiguous veins, inevitably leading to inferior vena cava (IVC) tumor thrombus (1, 2). The standard management of non-metastatic RCC with IVC tumor thrombus is surgical resection. Suprarenal cavectomy is indicated when bulky IVC tumor thrombus adheres significantly to the suprarenal IVC (2, 3).

Juxtarenal cavectomy is generally well tolerated in patients with preexisting obstruction and a right-sided RCC (2, 3). This is due to the fact that infrarenal IVC drains through lumbar veins which is enlarged in the setting of chronic venous obstruction. The left renal vein also drains through a lumbar vein, the left gonadal vein, and the left adrenal vein, in addition to neovascularization that may have occurred.

In the case of a left-sided RCC, where left nephrectomy and juxtarenal cavectomy is performed, right renal venous drainage poses a unique anatomic dilemma due to the lack of alternate pathways for venous drainage of the right kidney following juxtarenal cavectomy.

Here, we report a case of a patient with RCC who underwent left nephrectomy and juxtarenal cavectomy with reno-gonadal anastomosis for right renal venous drainage as a method of vascular reconstruction.

## Case Report

### Diagnosis

A 62-year-old male patient presented with hematuria and some limited worsening of chronic bilateral lower extremity edema, who was obese and had bilateral lower extremity edema. Cross-sectional imaging revealed a large left renal mass with a level II IVC tumor thrombus.

No overt metastatic disease was noted. Infrarenal IVC was noted to be atretic, and there was a concern for iliofemoral venous occlusion with extensive collateralization. A lower extremity ultrasound duplex showed extensive bilateral lower extremity deep vein thrombosis.

### Surgical management

The patient underwent open left radical nephrectomy, adrenalectomy, retroperitoneal lymph node dissection, and resection of juxtarenal IVC. Resection of IVC was for all areas concerning the tumor, extending from the atretic infrarenal IVC to the cranial tumor limit in the retrohepatic IVC. Ultrasound was used to verify the superior limit of the thrombus, and on opening the superior cava, no gross invasion was noted at the cranial caval resection margin. This superior IVC closure was completed with a four-layer 2-0 Prolene reconstruction after the stapler failed to fire due to desmoplasia. The caudal IVC resection was extended to a portion of infrarenal IVC which had no flow and which was presumed to be filled with bland thrombus. The caudal portion of the cava was closed with a two-layer 4-0 Prolene reconstruction.

Consistent with the preoperative CT, the right renal and the right gonadal veins were seen in close proximity and remained patent sonographically. It was decided that preserving this vascular connection for right renal venous drainage would be feasible even with an adequate cavectomy for tumor resection. During cavectomy, the lateral limited segment of IVC connecting the right renal vein and right gonadal vein was preserved intact. The medial aspect of this preserved venous wall was tubularized to form a reno-gonadal anastomosis that would serve as the primary drainage for the right kidney postoperatively. Some element of this drainage was hypothesized to have already been occurring, based on the preoperative computed tomography (CT) findings, due to chronic obstruction of the juxtarenal cava. The reconstruction was covered with an omental flap. Total blood loss was 4.5 liters and total intensive care unit (ICU) stay was 2 nights. Following an initial heparin drip, the patient was transitioned to apixaban and discharged in 7 days. Postoperative recovery was otherwise uneventful.

Surgical pathology was consistent with a 13.4 cm, grade 4, clear cell RCC with infradiaphragmatic IVC thrombus (pT3bN0). The tumor did not have rhabdoid/sarcomatoid features and surgical margins were negative. Around 0/17 lymph nodes were involved.

At 2-month follow-up, he had stable renal function (Figure 2) and improvement in his chronic bilateral lower extremity edema. He had no functional limitations. Anticoagulation was maintained, and he was free of recurrence at the 3-year follow-up. Estimated glomerular filtration rate (eGFR) trends are shown in Figure 3. Adjuvant therapy was not utilized as this patient was treated in an era that predated regulatory approval of adjuvant pembrolizumab for stage 3 clear cell RCC.

**Figure 1:**
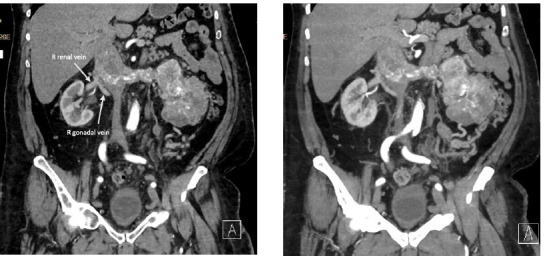
Preoperative right renal venous drainage. Preoperative coronary computed tomographic (CT) angiography of the abdomen and pelvis showing the right gonadal vein in close proximity to the right renal vein as well as tumor originating from the left kidney and suprarenal invasion of the inferior vena cava.

**Figure 2:**
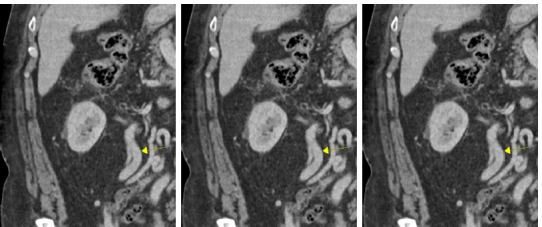
Right renal venous drainage 2 months status post nephrectomy. Coronal computed tomographic angiography of the abdomen and pelvis taken approximately 2 months post nephrectomy demonstrating right renal venous drainage through patent reno-gonadal anastomosis. Note that the yellow arrow in the figure refers to right renal venous drainage postoperatively.

**Figure 3:**
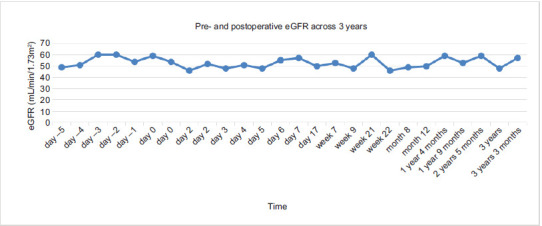
eGFR trends visualized through a histogram. Histogram demonstrating eGFR with time spanning from 5 days pre-operation to 3 years postoperation with the day of the operation being day 0.

## Discussion

In this article, we describe right reno-gonadal anastomosis as a method for right renal venous drainage following juxtarenal cavectomy for left-sided renal carcinoma. We believe the ideal patient for this option would be the patient with (i) preexisting caval occlusion from tumor thrombus resulting in collateralization of the right renal venous drainage, (ii) close proximity of the right renal and right gonadal veins, (iii) an uninvolved and short segment of cava that connects the right renal and gonadal veins such that anastomosis need not be circumferential, and (iv) an atretic infrarenal IVC with such a limited flow that a tube graft would likely thrombose and the retrograde flow into the infrarenal IVC alone would not be feasible. All of these criteria were present in the reported case.

Additional considerations are that (i) anticoagulation is likely beneficial in maintaining the patency of right renal venous flow, (ii) mobilization of the right kidney, ureter, and adrenal gland should be avoided to prevent disruption of additional venous collaterals, (iii) care should be taken to minimize any trauma to the right renal artery which sits just behind the juxtrarenal IVC, (iv) preoperative CT angiography is helpful in defining venous collateralization, and (v) adjuvant pembrolizumab which is an immunotherapy has been shown to reduce RCC recurrence in high-risk patients such as ours. According to the current risk stratification guidelines, adjuvant therapy would be indicated for this patient.

IVC resection without replacement is considered a standard option for the management of some patients with IVC thrombus (2, 5). Outside of the unique option employed in our patient, this generally involves retrograde flow into the infrarenal IVC or lumbar vessels. In one retrospective study by Du et al. (2020), seven patients diagnosed with RCC and an IVC tumor thrombus underwent a robotic left radical nephrectomy and segmental IVC resection without caval replacement or shunt reconstruction. Precise preoperative cavography was used to determine the degree of right renal vein and caudal IVC collateralization. Collateralization reconstruction was done utilizing CT-based three-dimensional reconstruction. No patients needed dialysis or died during the 3–6 months of follow-up; one patient developed liver metastasis 15 months postoperation (7). This study serves as an example of how IVC reconstruction following radical nephrectomy with high levels of collateral vasculature is a surgical treatment alternative to polytetrafluoroethylene (PTFE) graphs in patients with appropriate supporting vasculature.

Operative techniques for RCC depend on the degree of caval involvement and collateral venous drainage. However, each case is unique and nuanced and may require complex decision-making (8). Caval resection techniques include tangential, circumferential, and en bloc resections. Reconstruction can be done with venorrhaphy, patch grafting (patch cavoplasty), or interposition tube grafting, each coming with various complications (9, 6).

Generally, for cases with circumferential caval invasion where cavectomy is being considered, the alternative to not reconstructing the IVC will be an IVC tube graft. This can be done with a number of autologous or nonautologous options, but PTFE is most common in our practice. Bernike et al. (2014) conducted a retrospective study evaluating 26 patients that underwent radical nephrectomy with IVC resection and PTFE prostheses for IVC replacement. Of the 38% of postoperative complications, 19.2% of their patients had thrombosis of their grafts within the first year. Ciancio et al. (2021) studied four male patients who underwent surgical resection for RCC with tumor thrombus invading the IVC (4). The use of a ringed PTFE graft with an IVC filter within the graft for these patients appeared to be a safe vascular means of IVC reconstruction after surgical resection (4). At follow-up spanning 48 months, grafts remained patent and there was no occurrence of pulmonary embolism (4). Ultimately, although PTFE grafts reduce the risk of complication compared to other types of grafts, it still presents a fundamental risk of thrombosis.^1^

Patients with preexisting thrombus and limited blood flow in the infrarenal IVC are likely not good candidates for an IVC tube graft due to the potential for thrombus propagation and embolization. In our experience, this situation is very common when a tube graft is being considered. As a result, tube grafts are exceedingly rare in our practice. We find that most IVC resections are either (i) small enough to be closed primarily or with patch cavoplasty or (ii) large enough and coexisting with infrarenal IVC bland thrombus and limited flow such that a cavectomy without reconstruction is appropriate.

## Conclusion

Left-sided nephrectomy and juxtarenal cavectomy requires a different operative approach than the analogous right-sided operation. Reno-gonadal anastomosis is a feasible option for selected patients undergoing this operation. Alternative options for right renal venous drainage in this setting include a PTFE tube graft or drainage through preexisting collaterals like the infrarenal IVC (retrograde) or lumbar vessels (retrograde). This case highlights how reno-gonadal drainage is a feasible option in this setting.
